# Genome size variation in deep-sea amphipods

**DOI:** 10.1098/rsos.170862

**Published:** 2017-09-13

**Authors:** H. Ritchie, A. J. Jamieson, S. B. Piertney

**Affiliations:** 1 Institute of Biological and Environmental Sciences, University of Aberdeen, Zoology Building, Aberdeen AB24 2TZ, UK; 2 Oceanlab, University of Aberdeen, Newburgh, Aberdeenshire AB41 6AA, UK

**Keywords:** genome size, deep sea, amphipods, gigantism, adaptation, evolution

## Abstract

Genome size varies considerably across taxa, and extensive research effort has gone into understanding whether variation can be explained by differences in key ecological and life-history traits among species. The extreme environmental conditions that characterize the deep sea have been hypothesized to promote large genome sizes in eukaryotes. Here we test this supposition by examining genome sizes among 13 species of deep-sea amphipods from the Mariana, Kermadec and New Hebrides trenches. Genome sizes were estimated using flow cytometry and found to vary nine-fold, ranging from 4.06 pg (4.04 Gb) in *Paralicella caperesca* to 34.79 pg (34.02 Gb) in *Alicella gigantea*. Phylogenetic independent contrast analysis identified a relationship between genome size and maximum body size, though this was largely driven by those species that display size gigantism. There was a distinct shift in the genome size trait diversification rate in the supergiant amphipod *A. gigantea* relative to the rest of the group. The variation in genome size observed is striking and argues against genome size being driven by a common evolutionary history, ecological niche and life-history strategy in deep-sea amphipods.

## 1. Introduction

Understanding the causes and consequences of the extraordinary variation in genome sizes found among eukaryotes is an enduring issue in ecology and evolution. Genome sizes range from less than 2.3 Mb in the protist *Encephalitozoon intestinalis* [1] to over 149 Gb in the canopy plant *Paris japonica* [2]. Genomes can increase or decrease in size through a variety of mechanisms, including whole-genome duplication [3], the loss or gain of individual genes or gene families [4], recombination events [5,6] or the gradual accumulation of ‘junk’ genetic material such as pseudogenes [7], transposable elements (TEs) [8] or repeat arrays [9].

Despite a growing body of data on genome sizes from across a broad taxonomy, plus a recognition that genome size variation is at least partly determined by natural selection, there is still little consensus on the underlying ecological [10] or environmental [11] drivers of genome size. Indeed, the lack of any overarching phylogenetic signature for genome size, and the high variation that can occur within taxonomic lineages, suggests that genome size diversity is a consequence of multiple factors with varying influence in different taxa and in different habitats. Notwithstanding, multiple hypotheses have been proposed that attempt to provide a general explanation or single driver for genome size in particular taxonomic groups. The majority of focus has been on the interrelationships between genome size, nuclear envelope size, cell size and body size, and the ecological or physiological traits that can influence one or more of these individual components [12].

There are well-established positive relationships between genome size and nucleus size, and between nucleus size and cell size in all major taxonomic groups. It has been suggested that these are a consequence of large cells requiring large genomes for structural reasons, and larger cells necessitating larger nuclei to maintain efficient transport of mRNA into the cytoplasm [13]. Similarly, there are established links between key ecological, physiological and life-history traits and cell size that could have downstream effects on genome size evolution and diversity. For example, fast growth rate and high metabolic rates of species with r-selected life-history traits can select for small cell size through mechanical constraints associated with faster cell replication and metabolic activity, and/or a requirement to allocate phosphorous from DNA to RNA because of the demand for ribosomes to allow protein synthesis during rapid growth [14,15]. Both scenarios would then predict small genome sizes, which is indeed a characteristic feature of r-selected species [12,16].

These logical links between life history, cell size and genome size are readily observed in amphipod crustaceans. There is a 460-fold variation in crustacean genome sizes [17] and 100-fold variation within the amphipods [18], which reflects the wide range of marine, semi-terrestrial and freshwater habitats the taxa occupy. This has facilitated attempts to establish links between ecological or evolutionary constraint with genome size. For example, in several arctic species of amphipods, the reduced temperature lowers metabolic activity and growth rate relative to sub-polar and temperate regions. This would predict larger than average genome size and this has been largely shown to be the case [18]. Indeed, the largest crustacean genome is found in the arctic amphipod *Ampelisca macrocephola* [18].

Here we expand our understanding of the extent to which genome size can be considered a determinant of life-history, or vice versa, in Crustacea by providing, (to our knowledge), the first reports of genome sizes for deep-sea lyssianassoid amphipods collected from bathyal (1000–3000 m), abyssal (3000–6000 m) and hadal (6000 to approx. 11 000 m) depths. All aspects of the ecology and life history of the deep-sea amphipods would predict that they possess large genome sizes. In the classical r-K-A life-history continuum [19,20] the deep-sea amphipods are categorized as adversity or ‘A-selected’ species as a result of their low fecundity, slow development rate and by occupying ecological niches in poorly productive but predictable environments [21]. As with the arctic species, this would predict low metabolic rates, large cells and concomitantly large genomes.

Moreover, it has also been suggested that with the reduced temperature and increased hydrostatic pressure associated with greater ocean depth there should be an increase in cell size and life span in exactly the same way that Bergmann's principle generates the eco-geographical pattern of larger body size at higher latitude [22]. This would, in turn, also predict larger genome sizes in the amphipods relative to their shallow water equivalents. Indeed, this deep-sea extension of Bergmann's rule has been considered to generate the size gigantism characteristic of some deep-sea amphipods, notably in the genus *Eurythenes* [23] and more obviously the ‘supergiant’ amphipod *Alicella gigantea* [24] that can grow to lengths of over 25 cm.

The high hydrostatic pressures that define the deep-sea environment are also posited to drive large genome sizes [25,26]. It is well recognized that the deep sea has been colonized multiple times from shallower waters following climate-induced dysoxic mass extinction events [27,28]. The physiological effects of high hydrostatic pressure will limit the extent of range expansion of shallow water species, and those pioneer species that do reach the deep sea will have experienced an environmental stress that, among other things, disrupts the epigenetic control of TEs leading to TE proliferation and increased genome size [29–31].

Here we directly test the hypothesis that deep-sea amphipods have large genomes. We use flow cytometry to estimate genome sizes across 13 species of the Lysianassoidea from the Kermadec, Mariana and New Hebrides trenches and examine results in a phylogenetically controlled way to identify ecological and life-history correlates of genome size. We compare genome sizes with a broader range of amphipods, with particular recourse to key studies that have characterized genome sizes in arctic species [18] and across the depths of Lake Baikal [32].

## 2. Material and methods

### 2.1. Sample collection

Amphipods were collected over the course of three research cruises: in 2013 to the Kermadec Trench (approx. 26°43′ S 175°11′ W), New Hebrides Trench (approx. 21°13′ S 168°14′ E) and South Fiji Basin (approx. 24°58′ S 171°3′ E); and in 2014 to the Mariana Trench (approx. 18°49′ N 149°50′ E). In all cases an autonomous, full ocean depth rated lander vehicle was deployed to the seafloor at various depths (for details see table 1) which incorporated small baited funnel traps for sample collection [33]. Upon recovery of the lander vehicle, samples were frozen in liquid nitrogen and stored at −80°C.

**Table 1.** Table of 13 deep-sea amphipod species with calculated *C*-values, associated GenBank Accession Numbers and ecological data. (Ecological data were compiled from data in this study and wider literature.)

			GenBank accession numbers						
species	*C*-value ± s.e.	genome size (Gb)	16S	COI	18S	depth range (m)	max. depth (m)	median depth (m)	max. body length (mm)
*Lanceola* sp.	—	—	KP456062	KP713953	KT372894	—	—	—	—
*Abyssorchomene* sp.	9.81	9.59	KX034333	KX365238	KX365242	1010	2500	1995	9
*Abyssorchomene chevreuxi*	16.46	16.10	KX034329	KP713882	KP347454	3300	5400	3750	14
*Abyssorchomene distinctus*^a^	15.30 ± 0.04	14.96	KX034327	KP713886	KT372892	3800	6007	3004	14
Unidentified amphipod	9.09	8.89	KX034299	KX365239	KX365243	4984	7484	4992	—
*Alicella gigantea*^a^	34.79 ± 1.43	34.02	KX034290	KP713894	KP347467	5280	7000	4360	340
*Cyclocaris* sp.	4.73	4.62	KX034301	KP713899	KT372890	1907	6007	5034	15
*Eurythenes magellanicus*^a^	18.35 ± 0.74	17.95	KX034311	KP713957	KP347469	1229	5329	3486	85
*Eurythenes maldoror*^a^	18.86 ± 3.56	18.45	KX034310	KX365240	KX365244	3160	6230	4650	100
*Hirondellea dubia*^a^	4.74 ± 0.56	4.64	KX034251	KP713906	KP347459	6218	11, 000	7891	12
*Paracallisoma* sp.	19.54	19.11	KX034319	KX365241	KX365245	1726	5100	4237	28
*Paralicella caperesca*^a^	4.06 ± 0.54	3.97	KX034272	KP713921	KP347463	5925	7415	4453	18
*Paralicella tenuipes*^a^	4.13 ± 0.59	4.04	KX034284	KP713931	KP347464	4915	7415	4958	14
*Valettietta anacantha*	7.80	7.63	KX034322	KP713950	KT372893	6007	6007	3004	15

^a^Samples with replicates.

### 2.2. Phylogenetic reconstruction

Species were identified and phylogenetic relationships were ascertained based upon DNA sequence variation at two mitochondrial (COI and 16S rDNA) and one nuclear (18S rDNA) loci, according to Ritchie *et al*. [33]. In total, amphipod samples were sorted into 13 species and eight genera belonging to six families, all within the Lysianassoidea superfamily.

DNA sequence electropherograms were examined in MEGA v. 6.0.5 [34] and nucleotide alignments were made using webPRANK [35]. Individuals were identified to species or genus level using default parameters on NCBI BLASTn [36]. All species returned a 99–100% identity match to a BLAST hit with the exception of the unknown amphipod which returned positive matches to Lysianassoidea amphipods but without a high enough identity match to confidently assign it to either species or genus level.

For phylogenetic reconstruction the optimal evolutionary model for the dataset was identified using JModelTest 2.1.6 [37] using both the Akaike information criterion (AIC) and the Bayesian information criterion (BIC). Both AIC and BIC identified the general time-reversal substitution model (GTR + I + G) for COI and 18S rDNA, and the Hasegawa, Kishino and Yano model (HYK + G) as the best-fit model for the 16S rDNA dataset. Phylogenetic reconstruction was conducted using a Bayesian approach in *BEAST [38] where the analysis was given two runs each for 50 000 000 generations sampling 500 000 trees (every 100 generations) using the models of sequence evolution estimated by JModelTest but with the parameters estimated by *BEAST and *Lanceola* sp. was included as the outgroup. The first 150 000 trees were discarded as burn-in where the partition frequencies among the remaining trees gives the posterior probabilities to provide an estimate of clade credibility. Convergence of both runs was evaluated using Tracer v. 1.4.1 [39]. Trees were visualized using FigTree v. 1.4.2 [40].

### 2.3. Genome size estimation

Nuclear genome sizes were estimated using a flow cytometry approach where individual cell suspensions were prepared using a standard protocol [41] using whole amphipods in 1 ml of ice-cold Galbraith buffer [42]. Replicate estimates for individual species were conducted where possible (table 1). Owing to the high lipid content of deep-sea amphipods preparations were centrifuged at ×800*g* for 10 m to pellet cells and allow for the removal of the buffer suspension containing the unwanted lipids. Pelleted cells were re-suspended in 1 ml of phosphate-buffered saline (PBS) and stored at 4°C.

Chicken erythrocyte nuclei (CEN) from *Gallus gallus domesticus* were added to cell suspensions as an internal size standard and co-stained using propidium iodide at a final concentration of 50 ppm before incubation in the dark for 20 m at 4°C. Relative fluorescence of co-stained nuclei of samples were quantified using a FACSCalibur flow cytometer (BD Biosciences, USA) with an argon-ion laser emitting 15 mW of light at 488 nm. A minimum of 10 000 nuclei per sample were measured using Cell ProQuest software (BD Biosciences).

The relative fluorescence of nuclei peaks of interest were isolated using BD FACSDiva v. 7.0 software (BD Biosciences, NJ, USA). Haploid nuclear DNA content (*C*-value) of the samples were estimated from the fluorescence intensity of the sample and internal size standard using the haploid genome size of CENs which is 1.25 pg. *C*-values were subsequently converted into genome sizes using the standard conversion of 1 pg = 978 Mb as described in [43].

### 2.4. Statistical analysis

#### 2.4.1. Diversification rate analysis

To examine the patterns of diversification rate variation in genome size we used Bayesian analysis of macroevolutionary mixtures (BAMM) [44] and the R package BAMMtools [45]. Tree appropriate rate prior parameters were determined using the setBAMMpriors function in BAMMtools before two separate rjMCMC runs were conducted in BAMM. Each BAMM analysis was run for 10 000 000 generations where parameters were sampled every 50 000 generations and the first 100 000 generations were discarded as burn-in. MCMC runs were checked for convergence. The credible shifts and net diversification rates across the tree were computed using BAMMtools. It was not possible to account for incomplete lineage sampling as there are no diversity estimates available for Lysianassoidea amphipods although the samples investigated cover a good spread of the known diversity.

#### 2.4.2. Independent contrast analysis

Independent contrast analysis is used to transform phylogenetic information into independent values that can be used to detect co-variance between traits or variables of interest. Here Felsenstein's independent contrasts method [46] was used to examine correlations between genome size (pg), depth range (m), maximum depth (m), median depth (m) and maximum body length (mm) while controlling for the influence of phylogenetic signal. Phylogenetically independent contrasts were conducted using PDAP v. 1.07 (Phenotypic Diversity Analysis Package) [47] implemented within Mesquite v. 3.04 [48].

#### 2.4.3. Regression analysis

Linear regressions were also implemented to examine relationships between genome size (pg), maximum depth (m) and maximum body length (mm) across a range of amphipod species including those from the deep sea, arctic and Lake Baikal. Genome sizes, maximum depths and maximum body lengths for each species were collated from data collected in this study and taken from the wider literature (electronic supplementary material, table S1).

## 3. Results

### 3.1. Genome size estimation

Haploid genome size estimates for the 13 Lysianassoidea amphipod species examined in this study are presented in table 1. In total, genome sizes varied nine-fold from 4.06 ± 0.54 pg in *Paralicella caperesca* to 34.79 ± 1.43 pg in *A. gigantea*. The mean genome size across the species was 11.28 pg (11.03 Gb).

#### 3.1.1. Diversification rate analysis

**Figure 1.**
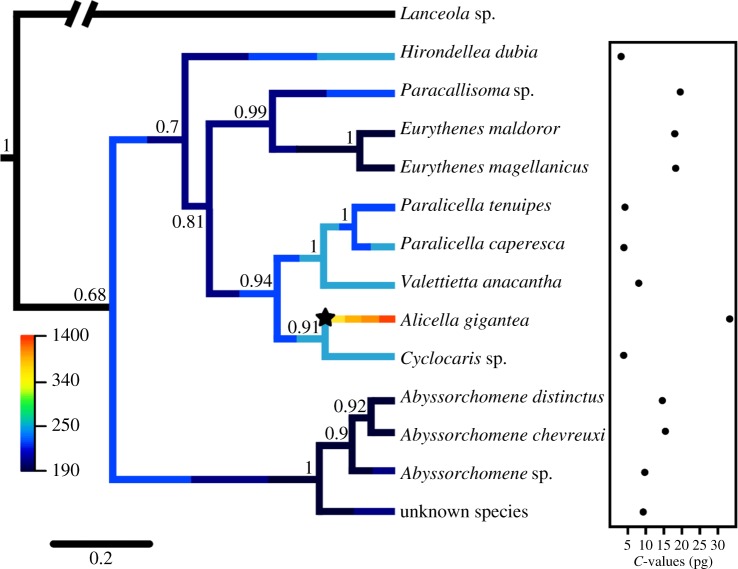
Mean phylorate plot for genome size evolution in Lysianssoidea amphipods using the BAMM MCC phylogeny is overlaid on the *BEAST phylogeny where branch colours indicate instantaneous rates of phenotypic evolution with rates divided into bins using the Jenks natural breaks method. The distinct shift configuration is indicated by a star where the shift was shown in the majority of the shift configurations sampled during simulation of the posterior (*f* = 0.59). Estimated *C*-values (pg) for the amphipods are also plotted.

All 13 species were successfully sequenced at 250 bp of the mitochondrial 16S rRNA gene, 627 bp of the COI gene and 599 bp of the 18S gene for a combined amplicon length of 1476 bp. GenBank accession numbers are provided in table 1 and a coalescent Bayesian tree is given in figure 1.

The BAMM diversification analysis reached a stationary state before 500 000 generations in both independent runs and using a Poisson prior (PP) of 1.0 it identified the most probable number of genome size diversification rate shifts was estimated as 1 (PP = 0.39) followed by 2 (PP = 0.21) and 0 (PP = 20). The mean phylorate plot shows an increase in mean diversification rate for genome size at the branch of the ‘supergiant’ *A. gigantea* (depicted as a star in figure 1). Individual rate-shift configurations sampled by BAMM were also investigated. The most probable scenario sampled showed a significant rate increase at the *A. gigantea* branch (PP = 0.59) and the remainder of the scenarios samples showed no significant rate changes across the phylogeny (PP = 0.41).

**Figure 2.**
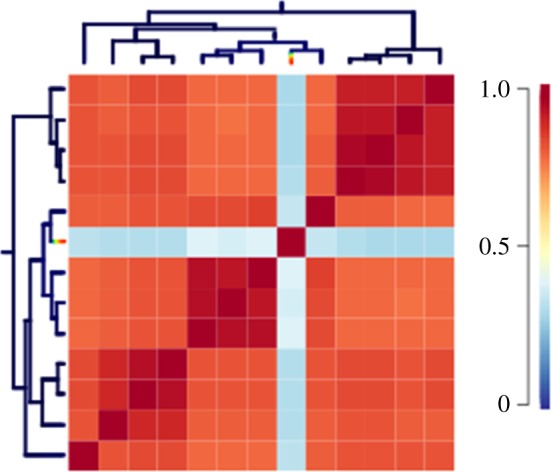
Macroevolutionary cohort matrix for genome size evolution in Lysianassoidea amphipods where each cell shows the pairwise probability that two species shares a common macroevolutionary rate regime. Pairwise probabilities are shown using a temperature scale from blue (*p* = 0) to red (*p* = 1). The BAMM MCC phylogeny is shown on the left and top of the cohort matrix for reference but the pairwise probabilities are calculated from a sample of trees from the posterior distribution of topologies and branch lengths.

A macroevolutionary cohort matrix of BAMM analyses for genome size evolution shows the pairwise probabilities that species share a common macroevolutionary rate regime (figure 2). There is a relatively high probability of shared rate regimes across the whole Lysianassoidea superfamily with the exception of *A. gigantea* which has a distinct macroevolutionary rate regime. Within the Lysianassoidea there are also groups that have higher than average pairwise probabilities of shared rate regimes. All species within the *Abyssorchomene* group have a high probability of shared rate regimes with each other and the unknown amphipod species which is likely to be another *Abyssorchomene* species given its phylogenetic placement. Both *Paralicella* species have a high probability of shared rate regimes with *Valettietta anacantha*. Both *Eurythenes* species also have a high probability of shared rate regimes between themselves and with *Paracallisoma* sp.

#### 3.1.2. Independent contrast analysis

Within the deep-sea amphipods a phylogenetically independent contrast analysis showed no strong or significant interactions between genome size and depth range (*r* = 0.063, *p* > 0.05), maximum depth (*r* = 0.019, *p* > 0.05) or median depth (*r* = 0.075, *p* > 0.05). A significant correlation was shown between genome size and maximum body length (*r* = 0.890, *p* < 0.001) however when the ‘supergiant’ amphipod *A. gigantea* was removed from the analysis the strength of this correlation was reduced (*r* = 0.504, *p* = 0.09).

#### 3.1.3. Regression analysis

The deep-sea amphipods do not have a larger genome size relative to other amphipods groups that have been studied (electronic supplementary material, figure S1). The average genome size of deep-sea amphipods was smaller than the arctic species (*t*_9_ = −1.18, *p* = 0.27) but greater than the freshwater Lake Baikal species (*t*_12_ = 2.80, *p* = 0.01). Arctic species also exhibited greater genome sizes than Lake Baikal species (*t*_7_ = 2.17, *p* = 0.07).

Overall the positive relationship between genome size and body size shown for the deep-sea amphipods is retained across all the amphipod species (*R*^2^ = 0.3067) (electronic supplementary material, figure S2). However, a positive relationship between genome size and maximum depth is also shown (*R*^2^ = 0.3409), though this is primarily driven by the amphipods from Lake Baikal (electronic supplementary material, figure S3).

## 4. Discussion

The salient finding of this study is that there is considerable genome size variation within the deep-sea Lysianassoidea amphipods. Among the 13 species examined there is a nine-fold change in genome size ranging from 4.06 pg (3.97 Gb) in *P. caperesca* to 34.79 pg (34.02 Gb) in *A. gigantea*. The mean genome size across the species was 11.28 pg (11.03 Gb). Given that the majority of all animal genomes recorded are less than 5 pg (4.89 Gb) and genomes over 10 pg (9.78 Gb) are considered to be large [49], then among these deep-sea amphipods analysed here there are six large genomes, five small genomes and two of intermediate size. Genome sizes for previously recorded amphipod species range from 0.94 pg (0.92 Gb) to 64.62 pg (63.33 Gb) with a mean genome size of 9.08 pg (8.89 Gb). This places the deep-sea Lysianassoidea amphipods at the larger end of genome size spectrum for amphipods.

As such, while there are clearly some large genomes among the deep-sea amphipods, large genome sizes are not a characteristic of the group. While their average genome size is larger than the amphipods in the freshwater Lake Baikal this is not true for the average genome size observed in arctic environments. This is sufficient to reject the hypothesis that genome size is determined by their common position in the r-K-A life-history continuum or an equivalent ecological niche in the extreme deep-sea environment.

It was predicted that the low temperature [22], high hydrostatic pressure [25,26] and A-selected life-history would select for large genome sizes across the group, but this is clearly not the case. Even among sympatric pairs of deep-sea species there is a large range of genome sizes suggesting an absence of a dominant environment variable influencing genome size. Moreover, independent contrast analysis found no relationship with depth which might have been expected if hydrostatic pressure was the primary selection pressure driving large genome sizes. Indeed, the patterns observed are somewhat counterintuitive given that the deepest living amphipod *Hirondellea dubia* has a small genome size of 4.74 pg at approximately 11 000 m whereas *Eurythenes magellanicus* has a genome size of 18.35 pg with a maximum known depth of 5329 m.

There was clear genome size rate diversification in genome size evolution across the group, with *A. gigantea* showing an enhanced rate of phenotypic evolution at a relatively derived position in the overall phylogeny. This is consistent with the conjecture that larger genomes are secondarily derived from smaller genomes [26], though this does not appear to be a consistent feature across the phylogeny, nor does it shed light on the processes that underpin genome size increase.

One clear pattern that was apparent both in deep-sea species and in the broader amphipod group was the positive relationship between genome size and body size [22,50]. The two genera that exhibit size gigantism had considerably larger genomes, with the giant amphipods *Eurythenes* spp. that reach maximum body sizes of 85 and 100 mm [51] having intermediate-large genome sizes of 18.35 and 18.86 pg, respectively, and the ‘supergiant’ amphipod *A. gigantea* with a maximum body size of 340 mm ([52] in [53]) having a large genome size of 34.79 pg. The majority of the Lysianassoidea are considerably smaller than *Eurythenes* and *Alicella* with average body sizes of approximately 9–14 mm and the majority of these having smaller genomes below 10 pg. If the largest species, *A. gigantea,* is removed from the analysis the relationship becomes marginal and certainly for the smaller bodied amphipods there is no clear relationship between body size and genome size. This is in accordance with the assertion that *A. gigantea* has an accelerated genome size diversification rate which is significantly distinct from the remainder of the Lysianassoidea.

Flow cytometry provides a rapid, economical and accessible approach for investigating genome size variation across taxa, but current data do not encompass a wide enough range of species and habitats, and it provides limited information on the changes in genome content and structure that drives the observed diversity [54]. Understanding the patterns and drivers of genome size evolution in deep-sea amphipods would benefit from the addition of phylogenetically similar shallow water counterparts to allow more extensive comparisons to be made. Another challenge moving forward is to establish whether the variation observed for the Lysianassoidea amphipods reflects changes in gene content, is a consequence of gene duplication or is influenced by the occurrence of TEs. Generally, the latter might be expected to be a major driver given the extreme and stressful environmental conditions associated with the deep sea. This might result in the increase of TEs associated with the disruption of epigenetic control [55–57]. Indeed, a growing body of genomic data available for *Paralicella tenuipes* [58] has shown both the presence of copia retrotransposons and evidence for several duplication events for two important heat-shock proteins [59]. Notwithstanding, the vastly accelerated genome size expansion shown in *A. gigantea* may also identify a whole-genome duplication event rather than solely being attributed to an accumulation of TEs, but this would require further investigation.

Overall, the occurrence of high genome size variation within a relatively small taxonomic group of deep-sea amphipods occupying an equivalent habitat and ecological niche emphasizes how problematic it can be to identify simple drivers of diversity, especially from correlative assessment. In all likelihood, the large variation in genome size will either be attributable to multiple factors acting in concert or, with different drivers operating in different taxonomic groups, habitats and in different times.

## Supplementary Material

Supplementary Table 1

## Supplementary Material

Supplementary Figure 1

## Supplementary Material

Supplementary Figure 2

## Supplementary Material

Supplementary Figure 3

## Supplementary Material

Flow Cytometry Data

## Data accessibility

All data necessary to reproduce the results in this paper can be found in the electronic supplementary material.
