# Supplementary material for: Genome size variation in deep-sea amphipods
Source: R Soc Open Sci. 2017 Sep 13;4(9):170862. doi: 10.1098/rsos.170862 (PMC5627123; doi:10.1098/rsos.170862)
Supplement: Supplementary Table 1 [file rsos170862supp1.docx]

**Supplemental Table 1. Table of all amphipod species with calculated C-values, genome size estimates, Genbank Accession Numbers and ecological data compiled from data in this study and wider literature.**

|  |  |  |  | Genbank Accession Numbers | | |  | | | |
| --- | --- | --- | --- | --- | --- | --- | --- | --- | --- | --- |
|  | **Species** | **C-Value ± SE** | **Genome Size (Gb)** | **16S** | **COI** | **18S** | **Depth Range (m)** | **Max. Depth (m)** | **Median Depth (m)** | **Max. Body Length (mm)** |
| Deep Sea | ***Lanceola* sp.** | - | - | KP456062 | KP713953 | KT372894 | - | - | - | - |
| Deep Sea | ***Abyssorchomene* sp.** | 9.81 | 9.59 | KX034333 | KX365238 | KX365242 | 1010 | 2500 | 1995 | 9 |
| Deep Sea | ***Abyssorchomene chevreuxi*** | 16.46 | 16.10 | KX034329 | KP713882 | KP347454 | 3300 | 5400 | 3750 | 14 |
| Deep Sea | ***Abyssorchomene distinctus*** | 15.30±0.04 | 14.96 | KX034327 | KP713886 | KT372892 | 3800 | 6007 | 3004 | 14 |
| Deep Sea | **Unidentified amphipod** | 9.09 | 8.89 | KX034299 | KX365239 | KX365243 | 4984 | 7484 | 4992 | - |
| Deep Sea | ***Alicella gigantea*** | 34.79±1.43 | 34.02 | KX034290 | KP713894 | KP347467 | 5280 | 7000 | 4360 | 340 |
| Deep Sea | ***Cyclocaris* sp.** | 4.73 | 4.62 | KX034301 | KP713899 | KT372890 | 1907 | 6007 | 5034 | 15 |
| Deep Sea | ***Eurythenes magellanicus*** | 18.35±0.74 | 17.95 | KX034311 | KP713957 | KP347469 | 1229 | 5329 | 3486 | 85 |
| Deep Sea | ***Eurythenes maldoror*** | 18.86±3.56 | 18.45 | KX034310 | KX365240 | KX365244 | 3160 | 6230 | 4650 | 100 |
| Deep Sea | ***Hirondellea dubia*** | 4.74±0.56 | 4.64 | KX034251 | KP713906 | KP347459 | 6218 | 11,000 | 7891 | 12 |
| Deep Sea | ***Paracallisoma* sp.** | 19.54 | 19.11 | KX034319 | KX365241 | KX365245 | 1726 | 5100 | 4237 | 28 |
| Deep Sea | ***Paralicella caperesca*** | 4.06±0.54 | 3.97 | KX034272 | KP713921 | KP347463 | 5925 | 7415 | 4453 | 18 |
| Deep Sea | ***Paralicella tenuipes*** | 4.13±0.59 | 4.04 | KX034284 | KP713931 | KP347464 | 4915 | 7415 | 4958 | 14 |
| Deep Sea | ***Valettietta anacantha*** | 7.80 | 7.63 | KX034322 | KP713950 | KT372893 | 6007 | 6007 | 3004 | 15 |
| Antarctic | ***Acanthostepheia malmgreni*** | 2.94±0.02 | 2.88 |  |  |  | 550 | 550 | 275 | 45 |
| Antarctic | ***Ampelisca macrocephala*** | 64.62±1.42 | 63.33 |  |  |  | 270 | 280 | 145 | 30 |
| Antarctic | ***Anonyx nugax*** | 27±0.71 | 26.46 |  |  |  | 170 | 175 | 90 | 40 |
| Antarctic | ***Arctolembos arcticus*** | 16.09±0.8 | 15.77 |  |  |  | 191 | 205 | 110 | 36 |
| Antarctic | ***Epimeria loricata*** | 13.49 | 13.22 |  |  |  | 231 | 411 | 296 | 40 |
| Antarctic | ***Rhachotropis aculeata*** | 7.16±0.18 | 7.02 |  |  |  | 715 | 775 | 441 | 45 |
| Antarctic | ***Stegocephalus inflatus*** | 50.91±1.65 | 49.89 |  |  |  | 255 | 400 | 288 | 47 |
| Antarctic | ***Themisto libellula*** | 3.97±0.15 | 3.89 |  |  |  | 249 | 249 | 125 | 46 |
| Baikal | ***Acanthogammarus brevispinus*** | 5.82 | 5.7 |  |  |  | 197 | 200 | 102 | 36 |
| Baikal | ***Acanthogammarus godlewskii*** | 8.32±0.25 | 8.15 |  |  |  | 50 | 230 | 205 | 50 |
| Baikal | ***Acanthogammarus victorii*** | 5.43 | 5.32 |  |  |  | 71 | 90 | 55 | 67 |
| Baikal | ***Baikalogammarus pullisc*** | 2.15 | 2.11 |  |  |  | 0 | 10 | - | 5 |
| Baikal | ***Brachiuropus grewingkii*** | 7.94 | 7.78 |  |  |  | 1200 | 1300 | 700 | 90 |
| Baikal | ***Brandtia latissima*** | 6.13 | 6.01 |  |  |  | 28 | 30 | 16 | 18 |
| Baikal | ***Carinurus bicarinatus*** | 7.24 | 7.10 |  |  |  | 0 | 200 | - | 30 |
| Baikal | ***Cornugammarus maximus*** | 5.94 | 5.82 |  |  |  | 0 | 121 | - | 67 |
| Baikal | ***Crypturopus inflatus*** | 4.93 | 4.83 |  |  |  | 0 | 150 | - | 16 |
| Baikal | ***Dorogostaiskia parasitica*** | 6.3 | 6.17 |  |  |  | 200 | 200 | 100 | 12 |
| Baikal | ***Echiuropus macronychus*** | 3.33 | 3.26 |  |  |  | 48 | 50 | 24 | 12 |
| Baikal | ***Eucarinogammarus wagii*** | 5.39 | 5.28 |  |  |  | 392 | 400 | 204 | 57 |
| Baikal | ***Eulimnogammarus cyaneus*** | 3.82±0.07 | 3.74 |  |  |  | 2 | 2 | 1 | 15 |
| Baikal | ***Eulimnogammarus czerskii*** | 5.69 | 5.58 |  |  |  | 17 | 25 | 17 | 30 |
| Baikal | ***Eulimnogammarus marituji*** | 3.77 | 3.69 |  |  |  | 30 | 30 | 15 | 20 |
| Baikal | ***Eulimnogammarus melanochlorus*** | 4.22 | 4.14 |  |  |  | 0 | 10 | - | 33 |
| Baikal | ***Eulimnogammarus similis*** | 4.72 | 4.63 |  |  |  | 0 | 26 | - | 15 |
| Baikal | ***Eulimnogammarus verrucosus*** | 6.10±0.29 | 5.98 |  |  |  | 20 | 20 | 10 | 36 |
| Baikal | ***Eulimnogammarus violaceus*** | 5.65 | 5.54 |  |  |  | 0 | 30 | - | 30 |
| Baikal | ***Eulimnogammarus vittatus*** | 3.95±0.06 | 3.87 |  |  |  | 30 | 30 | 15 | 18 |
| Baikal | ***Garjajewia cabanisi*** | 10.57±0.16 | 10.36 |  |  |  | 1237 | 1250 | 632 | 80 |
| Baikal | ***Gmelinoides fasciatus*** | 3.68 | 3.61 |  |  |  | 10 | 10 | 5 | 15 |
| Baikal | ***Hyalellopsis carinata*** | 5.85 | 5.73 |  |  |  | 0 | 40 | - | 10 |
| Baikal | ***Linevichella vortex*** | 4.71 | 4.62 |  |  |  | 0 | 10 | - | 7 |
| Baikal | ***Macrohectopus branickii*** | 4.2 | 4.12 |  |  |  | 250 | 250 | 125 | 38 |
| Baikal | ***Micruropus parvulus*** | 3.66 | 3.59 |  |  |  | 674 | 680 | 343 | 3 |
| Baikal | ***Micruropus wohli*** | 3.94±0.11 | 3.86 |  |  |  | 1 | 4 | 4 | 9 |
| Baikal | ***Odontogammarus calcaratus*** | 4.1 | 4.02 |  |  |  | 880 | 1080 | 640 | 30 |
| Baikal | ***Palicarinus puzylli*** | 5.64 | 5.53 |  |  |  | 0 | 200 | - | 50 |
| Baikal | ***Pallasea brandtii*** | 7.6 | 7.45 |  |  |  | 41 | 60 | 40 | 30 |
| Baikal | ***Pallasea cancellus*** | 4.56 | 4.47 |  |  |  | 51 | 52 | 27 | 70 |
| Baikal | ***Pallasea cancelloides*** | 5.21 | 5.11 |  |  |  | 177 | 178 | 90 | 25 |
| Baikal | ***Pallasea kessleri*** | 6.26 | 6.13 |  |  |  | 61 | 61 | 31 | 33 |
| Baikal | ***Parapallasea borowskii*** | 7.44 | 7.29 |  |  |  | 1166 | 1176 | 593 | 55 |
| Baikal | ***Poekilogammarus pictoides*** | 3.18 | 3.12 |  |  |  | 0 | 30 | - | 30 |
| Baikal | ***Sluginella kietlinskii*** | 16.63 | 16.30 |  |  |  | 0 | 630 | - | 81 |
| Other | ***Gammarus lacustris*** | 9.35 | 9.16 |  |  |  | 23 | 23 | 12 | 14 |
| Other | ***Crangonyx pseudogracilis*** | 6.68 | 6.55 |  |  |  | 12 | 12 | 6 | 9 |
| Other | ***Eulimnogammarus cyaneus*** | 3.81 | 3.73 |  |  |  | 2 | 2 | 1 | 15 |
| Other | ***Apohyale crassipes*** | 0.94 | 0.92 |  |  |  | 1 | 1 | 1 | 5 |
| Other | ***Apohyale prevostii*** | 1.89 | 1.85 |  |  |  | 1 | 1 | 1 | 8 |
| Other | ***Hyale crassipes*** | 0.96 | 0.94 |  |  |  | 20 | 20 | 10 | 6 |
| Other | ***Orchestia cavimana*** | 1.77 | 1.73 |  |  |  | 1 | 1 | 1 | 13 |
| Other | ***Orchestia gammarellus*** | 2.81 | 2.75 |  |  |  | 1 | 1 | 1 | 20 |
| Other | ***Orchestia mediterranea*** | 2.28 | 2.23 |  |  |  | 1 | 1 | 1 | 20 |
| Other | ***Orchestia montagui*** | 1.71 | 1.68 |  |  |  | 1 | 1 | 1 | 8 |
| Other | ***Platorchestia platensis*** | 1.86 | 1.82 |  |  |  | 1 | 1 | 1 | 14 |
| Other | ***Talitrus saltator*** | 2.2 | 2.16 |  |  |  | 1 | 1 | 1 | 4 |
