# Supplementary material for: Genome size variation in deep-sea amphipods
Source: R Soc Open Sci. 2017 Sep 13;4(9):170862. doi: 10.1098/rsos.170862 (PMC5627123; doi:10.1098/rsos.170862)
Supplement: Supplementary Figure 1 [file rsos170862supp2.docx]

**Supplemental Figure 1. Median genome sizes (Gb) of the three amphipod groups; deep sea (n=12), arctic (n=8) and Baikal (n=36).**
