# Supplementary material for: Genome size variation in deep-sea amphipods
Source: R Soc Open Sci. 2017 Sep 13;4(9):170862. doi: 10.1098/rsos.170862 (PMC5627123; doi:10.1098/rsos.170862)
Supplement: Supplementary Figure 3 [file rsos170862supp4.docx]

**Supplemental Figure 3. Log-transformed regression of genome size (Gb) and maximum depth (m) for 75 amphipod species including deep-sea, arctic and freshwater Lake Baikal species.**
